# Food-Pharma Convergence in Medical Nutrition– Best of Both Worlds?

**DOI:** 10.1371/journal.pone.0082609

**Published:** 2013-12-16

**Authors:** Tamar C. Weenen, Bahar Ramezanpour, Esther S. Pronker, Harry Commandeur, Eric Claassen

**Affiliations:** 1 Erasmus University Rotterdam, Faculty of Economics, Rotterdam, The Netherlands; 2 Department of Virology, Erasmus Medical Center, Rotterdam, The Netherlands; 3 Athena Institute, Vrije Universiteit, Amsterdam, The Netherlands; 4 Viroclinics, Rotterdam, The Netherlands; 5 Erasmus University Rotterdam, Faculty of Economics, Rotterdam, The Netherlands; 6 Nyenrode Business University, Breukelen, The Netherlands; University of Houston, United States of America

## Abstract

At present, industries within the health and life science sector are moving towards one another resulting in new industries such as the medical nutrition industry. Medical nutrition products are specific nutritional compositions for intervention in disease progression and symptom alleviation. Industry convergence, described as the blurring of boundaries between industries, plays a crucial role in the shaping of new markets and industries. Assuming that the medical nutrition industry has emerged from the convergence between the food and pharma industries, it is crucial to research how and which distinct industry domains have contributed to establish this relatively new industry. The first two stages of industry convergence (knowledge diffusion and consolidation) are measured by means of patent analysis. First, the extent of knowledge diffusion within the medical nutrition industry is graphed in a patent citation interrelations network. Subsequently the consolidation based on technological convergence is determined by means of patent co-classification. Furthermore, the medical nutrition core domain and technology interrelations are measured by means of a cross impact analysis. This study proves that the medical nutrition industry is a result of food and pharma convergence. It is therefore crucial for medical nutrition companies to effectively monitor technological developments within as well as across industry boundaries. This study further reveals that although the medical nutrition industry’s core technology domain is food, technological development is mainly driven by pharmaceutical/pharmacological technologies Additionally, the results indicate that the industry has surpassed the knowledge diffusion stage of convergence, and is currently in the consolidation phase of industry convergence. Nevertheless, while the medical nutrition can be classified as an industry in an advanced phase of convergence, one cannot predict that the pharma and food industry segments will completely converge or whether the medical industry will become an individual successful industry.

## Introduction

**Figure 1:**
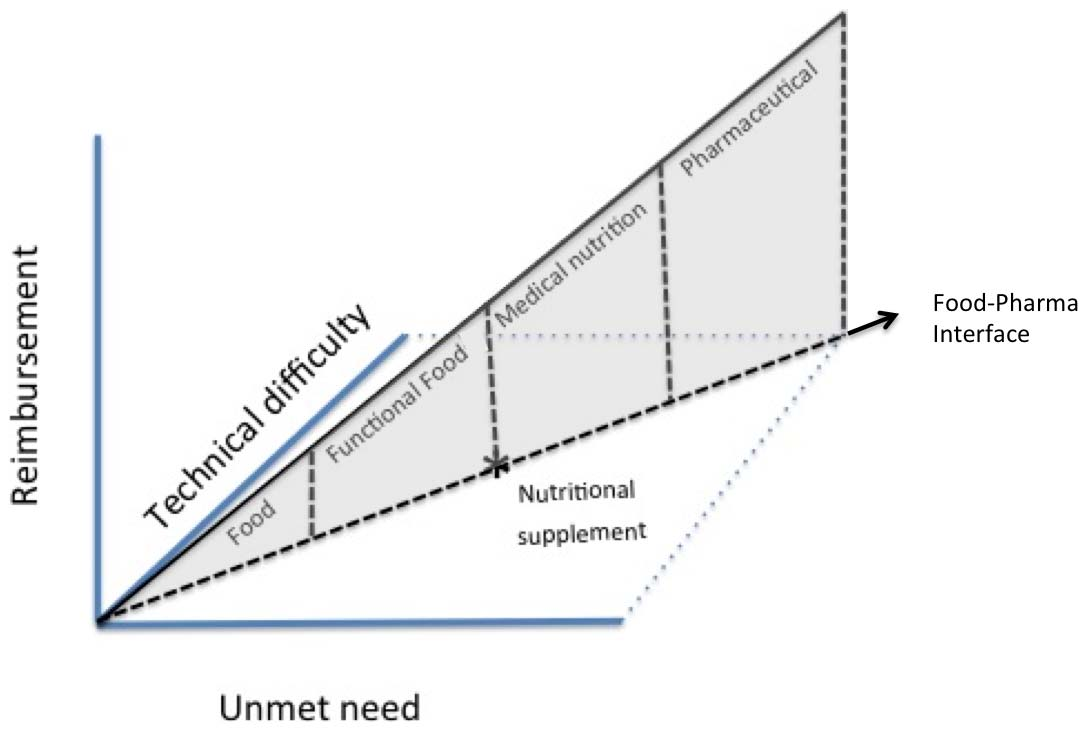
Industries situated at the food-pharma interface. Adapted from [7].

The Health & Life Sciences sector is currently undergoing significant change across all its industries. Boundary-crossing developments are occurring, especially between the food and pharmaceutical industries. The emergence of innovation at this intersection is blurring the clear boundaries between these two industries [1]. Such boundary-blurring innovation leads to industry convergence, which in turn results in the emergence of new industries. Food-pharma products resulting from this convergence are known as Nutritional Supplements (NS), Functional Foods (FF), and Medical Nutrition (MN). NS include vitamins, minerals, herbs, amino acids, and other related products intended to supplement the nutritional content of the diet in tablet/capsule dosage [2]. FF are conventional foods with added nutrients that claim to improve health beyond the basic nutritional functions [3]–[8]. MN products are specific nutritional compositions for disease intervention that effectively contribute to the therapeutic regimen by improving a patient’s general condition [9], [10]. MN can be divided into tube feeding and oral nutritional supplements (e.g. Nutridrink; Ensure; and Resource) and are primarily prescribed by healthcare professionals. NS, FF, and MN are food substances that are considered to improve health, and exist between conventional foods and pharmaceuticals at the so-called food-pharma interface (Figure 1) [11]. Nevertheless, the individual pharmaceutical and food companies recognize the risks in developing food-pharma inventions[10], [12]. They fear that the commercialization of boundary-spanning products [7] could result in a lower customer acceptance due to the ambiguous identity of the product [7].

The present study focuses on the emerging MN industry, where industry boundaries are still relatively undefined. This is reflected by the terminology used to describe this product category, which is most often perceived as confusing. MN is just one term among many others to indicate the same product category (e.g. oral nutritional supplement, medical food, clinical nutrition, enteral nutrition).

The European (EU) MN industry comprises 5 leading companies and currently finds itself in the growth phase of the industry lifecycle [7], [13]. It is difficult to predict the prerequisites for determining the future success of an emerging industry such as the MN industry, nonetheless: carefully categorizing industries and identifying industry boundaries is crucial and can lead to better consumer perception and higher market acceptance [7], [14]–[18]. In the view that millions of patients are suffering from disease-related malnutrition, including a surprisingly high proportion living in the developed countries/high income economies [7], [10], [19] and many studies have proven that nutritional interventions prevent and/or support the development of disease-related malnutrition [10], [19], MN is considered of high societal value. Therefore, defining industry boundaries may also have an indirect societal impact. The first step in identifying industry boundaries is by determining the status of industry convergence and thereby investigating how and which distinct industry domains have contributed to establish an industry.

**Figure 2:**
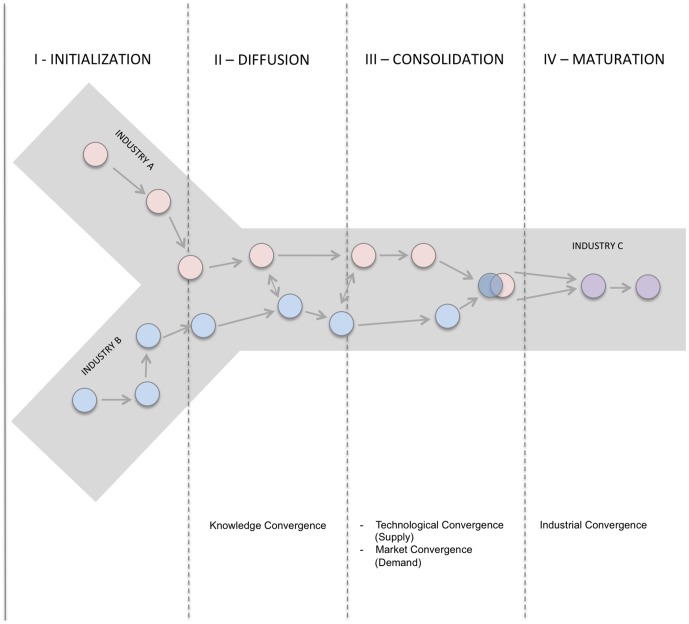
Linear model of convergence adapted from [20]–[22],[65].

In this research the concept of MN industrial convergence is based upon the assumption that the phenomenon of industry convergence proceeds along an evolutionary trajectory consisting of four phases: Initialization; Knowledge Diffusion; Consolidation; and Maturation (Figure 2) [20]–[22]. Such industry convergence has been observed in many industries such as telecommunications, computing and consumer electronics or cosmetics and pharmaceuticals [23]–[26]. In the initial stage, R&D of two or more distinct industries segments remains independent. It is during the knowledge diffusion stage where cross-disciplinary citations may eventually result into joint research collaborations (consolidation stage). As the metaphorical distance between the two knowledge areas decreases, technology development follows, which in turn leads to technology convergence [22]. It is believed that market convergence is also a consequence of the new technological combinations. Ultimately, sectors begin to merge with one another, completing the industrial convergence process.

This study shows how and which distinct industry domains have contributed to establish the MN industry. First we determine the extent of knowledge diffusion within the MN industry, subsequently we define the consolidation into the MN industry on the basis of technological convergence (Fig. 2), and eventually we identify the MN core domains and chart the technology interrelation and its influence on the MN industry development. Both knowledge diffusion and technological convergence are two important drivers of innovation and recognized as crucial components for industry growth [26], [27]. Specifically within the health and life science sector, both drivers contribute to the evolution of young and emerging industries such as the MN industry [28]. Moreover, scientific advancements are the key ingredient in stimulating both knowledge diffusion and technological convergence. The former - knowledge diffusion - is defined as the process through which knowledge is spread along a specific path in a social system [29]. Technological convergence implies a technological change where inventions emerge at the intersection of established and clearly defined industry boundaries [30]. The cumulative effect of both drivers ultimately leads to industry convergence [31].

The quantitative diffusion and consolidation results from this study will contribute to detailed insights in MN industry development and can help industry players to address specific innovation strategies for the future.

Patents have been proven to be a valuable source of information in mapping MN industry development [32], [33], they contain about 80% of all technological knowledge and are generally regarded as precursors of technological developments [22], [34]. In addition, they can be independently accessed and analyzed through various types of comprehensive and open databases [35]. Finally, in contrary to other knowledge sources, such as scientific literature, patents are categorized according to multiple technology classes according to their technological characteristics. This allows for accurate co-classification analyses to identify the interrelation between technologies [36]. Therefore, in this study, patent data was used to identify the evolutionary (technological) development of the MN industry.

## Methodology

**Figure 3:**
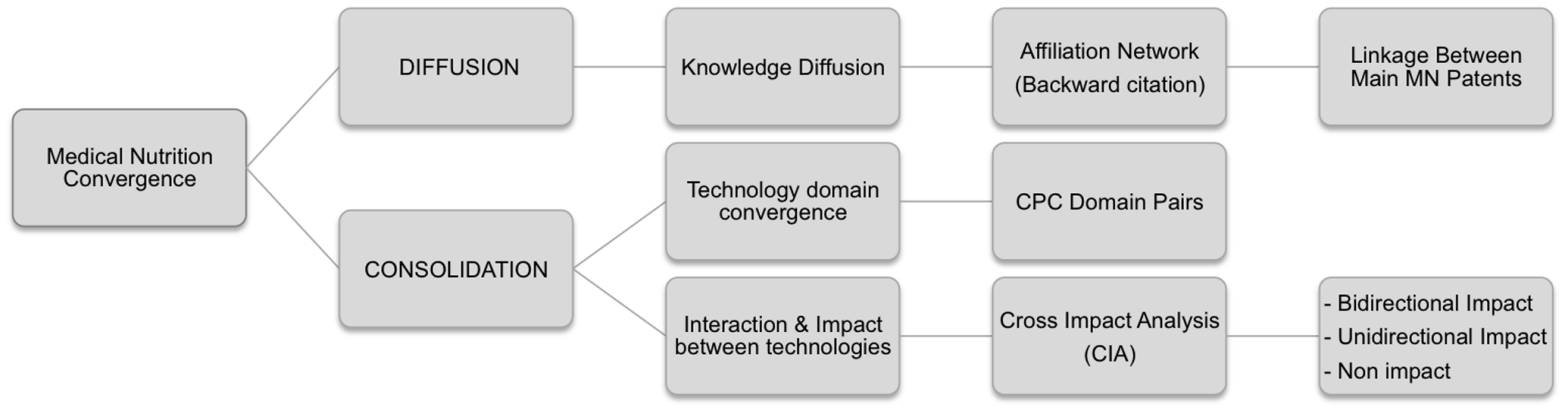
Research framework.

The methods applied in this study are based on research methods by Karvonen, Tseng, and Choi [30], [37], [38] and adapted to fit our research objective. To determine the stage of convergence in the MN industry, this study is divided into Knowledge Diffusion and Consolidation. Furthermore, the consolidation is divided into technological convergence, and CIA (Figure 3). Data on patents concerning MN was extracted from the Derwent Innovations IndexSM and Espacenet pertaining to the European published patent applications. In total, 274 patent applications were filed by the 5 leading EU MN companies from 1984 up to 2013 (so called; main patents).

### Knowledge diffusion

Since knowledge convergence is the first stage of convergence, the analysis of knowledge flow within the MN industry is an appropriate method for identifying possible current and future convergence between knowledge disciplines originating from different industries [39]. Patent citation data is considered an important information source for analyzing science-based knowledge flows. Patent citations within the MN industry are indicative for the technological relationship between patents in the MN industry [40]–[44]. Patent citations refer to the number of cited patents within the original patent application as an indicator of prior art. Such an analysis provides information of inter- industry competition and knowledge spillovers [30], [45].

In order to identify the knowledge diffusion within the MN industry, the backward citations of all main patents were extracted. Subsequently, we constructed an affiliation network visualizing the interrelations of all main patents of the European MN companies. This method is a powerful tool to analyze knowledge flows and within-industry competition [37]. The mutual linkage between the main MN patents were explored and visualized using the statistical software programs Ucinet and NetDraw [46]. This network represents the knowledge flows between the European MN companies (anonymous) and gives an indication of within industry competition.

### Technological convergence

In general, patents have multiple technology classifications depending on their claims. Since patents are classified into certain technological classes according to their technological characteristics, co-classification analysis identifies the interrelation between technologies [36]. The co-classification analysis measures the frequency by which two classification codes are jointly assigned to a patent and can be interpreted as an indication of the strength of the technological relationships. Ultimately, this allows for calculating technological convergence [35], [47]. The co-classification in this study is based on the Cooperative Patent Classification (CPC) codes [48]. Since the MN industry is not yet assigned to one specific classification category, the co-classification of different technologies currently delineates this industry. This is in accordance with the fact that the MN industry is still in growth phase as described earlier [7].

The expert designated CPC codes from each patent were extracted to analyze science-based technological convergence within the MN industry. CPC is an extension of the International Patent Classification (IPC) and is a joint endeavor of the European Patent Office (EPO) and the United States Patent and Trademark Office (USPTO) to harmonize the classification systems into a single system. This jointly developed classification system is much more granulated than the IPC system.

The CPCs were extracted from the patent search and analysis software ACCLAIMiP and the Espacenet portal. Since patents can be classified into several CPC groups, the co-classification provides information concerning technological convergence. In order to reveal the technological convergence domains within the MN industry, the first two (converging) CPC codes were extracted from all main patents and grouped into various domain combinations [30], [49], [50]. CPC codes are a hierarchical way of assigning the category to which every patent belongs [51]. The MN patents are categorized into classes, which are divided into sub-classes, main groups and sub-groups. The main groups are merged into domain combinations as illustrated in Table 1. In this study we make no difference between the orders of category combinations (e.g. no difference between 1–2 and 2–1). Subsequently, the number of patents per domain combination was divided in time blocks of 5 years, showing the evolutionary development of the emerging MN industry.

**Table 1:** Predominant CPC groups in MN patent literature.

Nr.	CPC Code	Groups
**1**	A23K1	Animal feeding-stuffs
**2**	A23G1	Cocoa; Cocoa products
**3**	A23F5	Coffee; Coffee substitutes; Preparations thereof
**4**	A61K8	Cosmetic or similar toilet preparations
**5**	F24D19	Details
**6**	A23D7	Edible oil or fat compositions containing an aqueous phase
**7**	Y02B30	Energy efficient heating, ventilation or air conditioning
**8**	A61J15	Feeding-tubes for therapeutic purposes
**9**	A23V2002	Food compositions, function of food ingredients or processes for food or foodstuffs
**10**	A23L1	Foods or foodstuffs
**11**	C07K16	Immunoglobulins
**12**	A61K9	Medicinal preparations characterized by special physical form
**13**	A61K45	Medicinal preparations containing active ingredients
**14**	A61K2039	Medicinal preparations containing antigens or antibodies
**15**	A61K33	Medicinal preparations containing inorganic active ingredients
**16**	A61K35	Medicinal preparations containing materials or reaction products thereof with undetermined constitution
**17**	A61K31	Medicinal preparations containing organic active ingredients
**18**	A61K38	Medicinal preparations containing peptides
**19**	A61K36	Medicinal preparations of undetermined constitution containing material from algae, lichens, fungi or plants, or derivatives thereof
**20**	A23C9	Milk preparations; Milk powder or milk powder preparations
**21**	A23C11	Milk substitutes
**22**	A61K2300	Mixtures or combinations of active ingredients
**23**	A23L2	Non-alcoholic beverages; Dry compositions or concentrates therefor
**24**	A23J1	Obtaining protein compositions for foodstuffs; Bulk opening of eggs and separation of yolks from whites
**25**	A23D9	Other edible oils or fats
**26**	C07K14	Peptides having more than 20 amino acids; Gastrins; Somatostatins; Melanotropins; Derivatives thereof
**27**	A23J7	Phosphatide compositions for foodstuffs
**28**	C12P19	Preparation of compounds containing saccharide radicals
**29**	C12P17	Preparation of heterocyclic carbon compounds with only O, N, S, Se or Te as ring hetero atoms
**30**	A61Q19	Preparations for care of the skin
**31**	A23L3	Preservation of foods or foodstuffs, in general
**32**	C12R1	Processes using micro-organisms
**33**	A23G3	Sweetmeats; Confectionery; Marzipan; Coated or filled products
**34**	A23F3	Tea; Tea substitutes; Preparations thereof
**35**	A23C21	Whey; Whey preparations
**36**	A23J3	Working-up of proteins for foodstuffs

There is a predicted lag in the convergent domains since patent applications are available in the public domain only 18 months after filing. As a result, the dataset is accurate to Jan 2012 and therefore by definition no 2013 patent applications could be included.

### Cross impact analysis

The identification of the overall structure of technologies and interaction among them is essential to recognize the maturity of technological trends and discover technological possibilities through convergence between various fields of technologies [52]. Cross Impact Analysis (CIA) is considered a reliable quantitative methodology to identify the core technologies and interrelations between technology domains [53]–[55] based on patent classification data [38]. In our study, the technology impact between various MN technology domains is analyzed based on patent co-classification data as described in technological convergence section. The impact between technologies can be derived from the CPC codes of the patent. Moreover, the impact of (A, B) can be defined as conditional probabilities between two technologies [38]. This means that the cross impact of technology A on technology B can be defined as follows: *Impact (A, B) = P (B|A) = (N (A ∩ B))/(N (A))*

In this equation, N (A) refers to the total number of patents included in domain A, and N (A∩B) indicates the number of patents, which include both domain A and domain B. The patent-based cross impact between domains can be analyzed by calculating the conditional probability with the number of patents in the patent classes. The score of index ranges from 0 to 1. If the score is close to 1, then technology domain A has a high impact on technology domain B and when the score is approaching the 0, the impact is considered lower.

Technology pairs based on the cross impact scores can be classified into three groups. In case 1, the so-called bidirectional impact, most of the patents in technologies A and B overlap; hence, both Impact (A, B) and Impact (B, A) are high. Consequently, conditional probabilities are relatively high and the impacts of one technology on the other technology are both high.

In case 2, called one directional impact, a high number of patents in technology A is also included in technology B, however, the portion of patents in technology B that is also included in technology A is relatively small. This means that Impact (A, B) is high, but Impact (B, A) is low. In this case, the impact between technologies A and B is unidirectional.

In case 3, called nonimpact, technologies A and B are almost exclusive and there is little interaction between them. Basically, these two technologies can be said to be almost independent.

Moreover, the individual impacts between the domains are visualized by means of network analysis depicting the type of interaction (arrow) between the domains (node). The direction of the arrow indicates the direction of impact between two domains. It visualizes whether technologies are equally influencing one another (bidirectional) or whether the impact of the first technology on the second is different from the impact of the second technology on the first (unidirectional) [38], [56].

Patent data is a valuable source of information and is useful in the study of technological convergence and diffusion as well as in technology interrelation and development. Nonetheless, not all inventions are patented and changes in patent law over the years make it difficult to analyze trends over time [57]. Since the protection afforded to patentees worldwide has been improved, the companies are more inclined to file for a patent than before [57]. Additionally, since CPC is a joint endeavor of the EPO and USPTO, this classification system is more detailed, up to date, and dynamic [51]. Subsequently, we have applied the quantitative patent-based CIA method of Choi [38] as opposed to the more conventional qualitative (CIA) approach, by means of literature surveys and expert interviews, aiming to overcome inconsistent outcomes. Furthermore, the citations lag between the application or grant year of the citing patent and that of the cited patents make it impossible to assemble all the main patents within the MN industry up until present time [58]. To address this limitation, a prediction line was drawn (result section *CIA)*.

## Results

In total, 274 patent applications were filed by the 5 leading European MN companies between 1984 and 2013. The MN patents can be assigned to 5 classes which are subsequently divided into 7 sub-classes , 37 main groups ,and 151 sub-groups.

### Knowledge Diffusion

**Figure 4:**
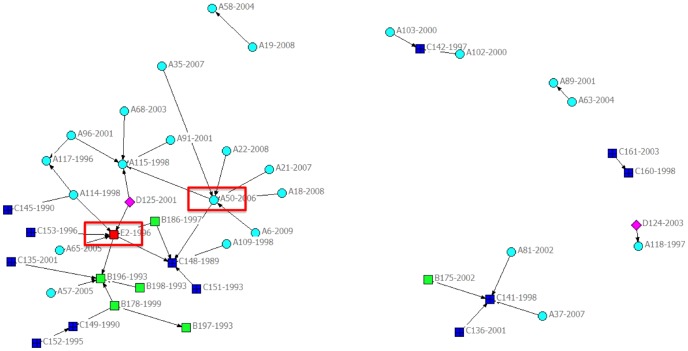
Knowledge diffusion within the MN industry - Network of the main patents (coded company, patent number - application year). Visualization presents the backward citing between main patents of MN companies. This network visualizes those patents that are linked. Symbols indicate the 5 MN companies; The direction of the arrow indicates the cited patent.

The knowledge diffusion network shows that most patents (78%) are not interrelated within the MN industry by means of patent citations. Interestingly, figure 4 shows that the remaining 22% of the patents lead back to two patent precursors and the CPCs of the precursors indicate convergence between the *Food – Food Compositions* and the *Food – Pharmaceutical Organic Active Ingredients* industrial domain combinations (Figure 4). The remaining 78% of the main patents are not linked to patents within the MN industry domain and are therefore linked to patents from other industrial domains. The high occurrence of patent linkage beyond the industrial domain indicates boundary-spanning convergence is taking place in MN development.

### Technological convergence

**Figure 5:**
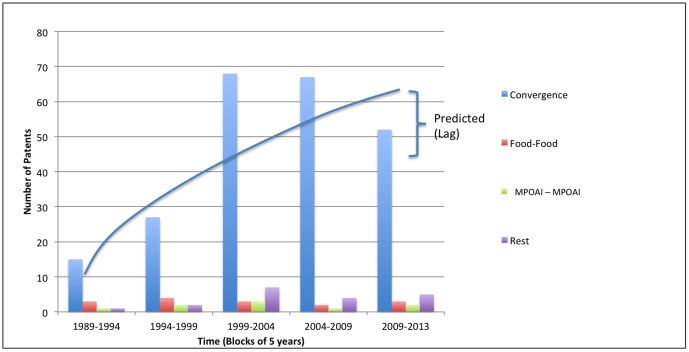
Evolution of single domains versus different domain convergence in MN – MN cannot be classified as a single domain but predominantly as a convergence between different domains.

**Figure 6:**
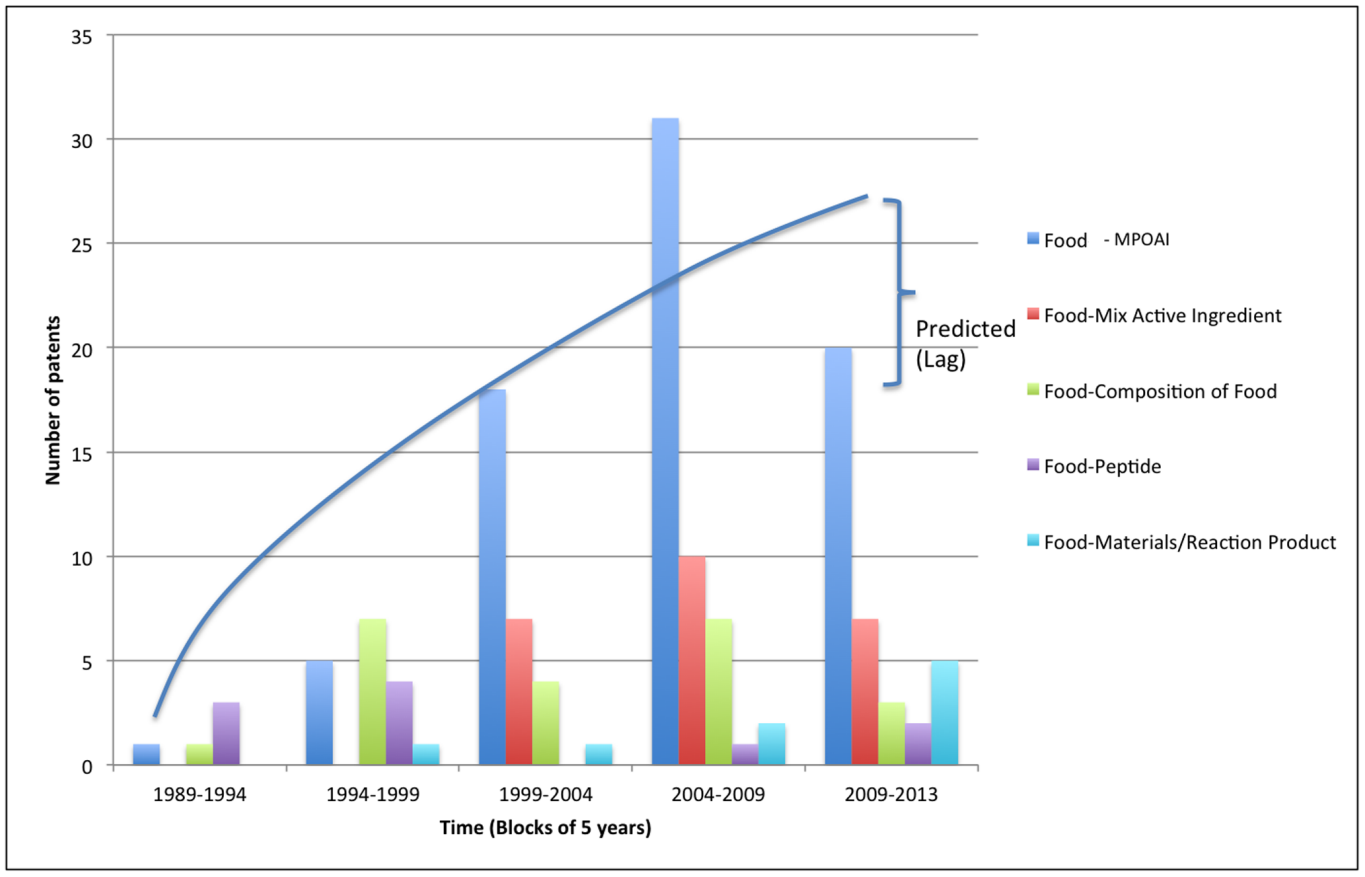
Food-Pharma dominates the domain convergence in MN - Selection of top 5 converging domains from 1989 up to 2013 in MN.

Figure 5 illustrates that between 1989 and 2013, 84% of all MN main patents show convergence between different industrial domains indicating technological convergence. Furthermore, figure 5 demonstrates that convergence of industry domains have played an essential role in the MN industry development since 1989, nevertheless, the importance of specific domain combinations varies over the course of time (Figure 6).

Further sub-categorization of the MN domains, indicating domain convergence, reveals the 5 most prevalent sub-groups: Food – Medicinal preparations containing organic active ingredients; Food – Medicinal preparations containing peptides; Food – Food Compositions; Food – Medicinal preparations containing combinations of active ingredients (MPOAI); and Food – Materials/Reaction Products (Figure 6).

Figure 6 shows that from 1989 until now Organic Active Ingredients, Food Compositions, and Peptide Compositions have played an essential role in the development of MN industry. In 1994 a new domain combination emerged: *Food – Materials/Reaction Product.* Since 1999, another new domain combination emerged: *Food – Medicinal preparations containing peptides.*

The principal domain convergence has occurred between the *Food* domain and *MPOAI* domain. Examples of MPOAI are: carbohydrates; sugars; carboxylic acids; hydrocarbons; amino acids; vitamins; and medicinal plant derivatives.

### CIA

**Figure 7:**
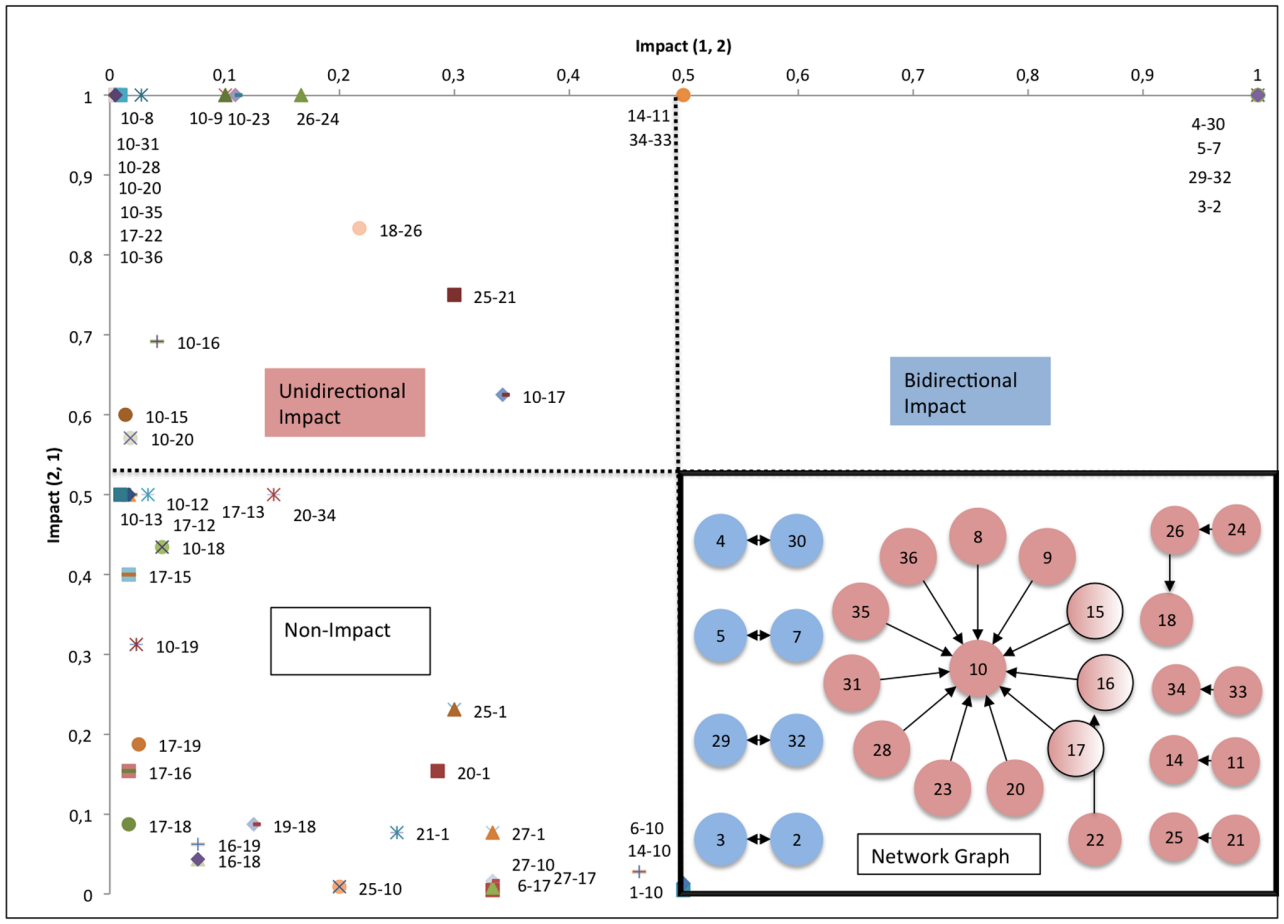
Grouping of the technology pairs in the MN industry, Network graph of bidirectional and unidirectional impact within the MN industry (1984–2013).

The cross impact scores help classify each technology pair into three groups: Bidirectional, Unidirectional, and Non-impact. The CIA network illustrates that 22 out of 47 technology pairs can be classified as bidirectional- or unidirectional impact (Figure 7).

Furthermore, the bottom-right quadrant of figure 7 illustrates a network graph of the relationships between technology domains within the MN industry. Each node represents a technology domain and the color of the node indicates its corresponding score that classifies the impact between two technology domains. The bidirectional impact technology pairs are expressed as blue nodes and the unidirectional impact technology pairs as red nodes. Furthermore, the direction of the arrows indicates the direction of impact. The network graph helps us identify the influencing- and influenced technology domains.

The network graph indicates that 11 technology domains directly influence the food domain. Eight of the eleven influencing domains originate from food (8, 9, 20, 23, 28, 31, 35 and 39) whilst three domains (15,16 and 17) originate from pharma. The domains impacting the food domain (10) that originate from pharma account for 138 patents, while the domains originating from food account for 57 patents.

The central positioning of Food (10) in the network graph shows that this technology domain can be considered as the core MN domain. Additionally, technological development from the pharmaceutical domain, especially medicinal preparations containing: inorganic active ingredients, organic active ingredients and materials or reaction products thereof with undetermined constitution, influence the core MN domain.

## Discussion and Implications

This study proves that the MN industry is a result of a bona fide food-pharma convergence. Additionally, the results indicate that the industry has surpassed the knowledge diffusion stage of convergence, and is currently in the consolidation phase of industry convergence. Nevertheless, while the medical nutrition can be classified as an industry in an advanced phase of convergence, one cannot predict that the pharma and food industry segments will completely converge or whether the MN industry will reach a state of maturation and become an individual successful industry. This confirms previous research which revealed the MN industry to be in the relatively early development stage of the technology life cycle [9]. The knowledge flows and subsequently trans-disciplinary technological convergence between the food-pharma technology domains have fine-tuned the MN industry as it is today. This study further reveals that although the MN industry’s core technology domain is food, the technological development is mainly driven by pharmaceutical technologies.

Although not scientifically proven, in the past few years literature has stated that the gap between pharmacology and nutrition science has been narrowing, a development stimulated by both disciplines [10]. The increase in technological convergence between food and MPOAI confirms this observation, which previously has been termed as “pharmaconutrition”. Although in the past only drugs were considered pharmacologically active substances, this new treatment paradigm embraces the fact that nutrients can have profound effects on immunological, metabolic and other pathophysiological processes of diseased patients [10], [59].

Our results show that there are currently five different CPC combinations required to define MN in patent literature. This emphasizes the necessity for a specific CPC code to clearly categorize MN, which may contribute to clearly delineating MN industry boundaries. Having its own identity may lead to better consumer perception and higher market acceptance thereby stimulating MN market growth.

Considering that convergence drastically alters industry structures, companies should consider evaluating whether their activities may be affected by trends of convergence [60]. By monitoring convergence trends, companies can benefit by commercializing on trans-disciplinary opportunities. The MN industry can be characterized as a convergent/converging area at the food-pharma interface and it is therefore crucial for MN companies to effectively monitor developments within as well as across industry boundaries. Both in the food- and pharmaceutical industry trends should be monitored, as our results indicate that critical knowledge is also developed in those fields [60]. Especially the technological development within the pharmaceutical industry is essential since our CIA results shows that pharmaceutical technologies have the greatest impact on MN development.

The knowledge diffusion results indicate a high occurrence of patent linkage beyond the MN industrial domain implying that the first step in boundary-spanning industry convergence: knowledge diffusion, is taking place in MN development. Our empirical analysis further reveals both knowledge and technological convergence between the food-pharma technological domains, thereby showing the first three phases of convergence of the linear model of convergence in the MN industry.

Nevertheless, it is often argued that factors other than technology are involved in the process of industry convergence. Weaver (2007) and Karvonen & Kassi (2013) believe that technology and industry convergence are often intrinsically linked, yet these two concepts are causally and conceptually distinct [30], [61]. Examples of those factors include: regulation, quality standards, business model innovation, changing customer requirements and industry channel structure. The process of food-pharma convergence is nurtured by the trend of regulatory convergence with respect to costly clinical research increasingly required for MN. These factors can be divided into supply (science, technology) and demand (consumer needs) factors.

**Figure 8:**
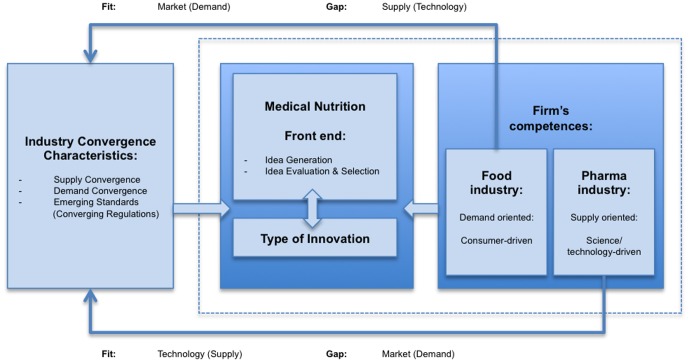
Front end of innovation activities in converging industries. Adapted from [26].

The absence of competencies in either supply or demand understanding may lead to considerable problems at the front end of innovation (idea generation, evaluation and selection) [26]. Our results indicate that the front end of MN innovation is affected by convergence (Figure 8). Especially as the process of innovation requires the combination of new knowledge and competencies owned by different industries domains [26]. Perhaps this is one of the reasons that the MN industry may currently be facing an innovation cliff [7], [9], [62]. We would argue that in the MN industry, front-end innovation challenges are related to the converging industries. For example; the food industry counterparts of the trans-disciplinary venture might experience challenges on the technological/supply aspect of the convergence (e.g. clinical trials (endpoints, quality standards, pharmacokinetics and pharmacodynamics [10]) whereas the pharmaceutical participant may find the consumer/demand experience (e.g. taste, texture, tolerance, smell) a particular bottleneck (Figure 8). Successful convergence would therefore require awareness on matching skills, experience and resources that would complement the, otherwise lacking, absorptive capacity [26]. Innovation managers must be aware of competence gaps on the supply and/or demand side. One way to bridge this gap is to identify external partners, already at the idea generation phase, with the additional competences to account for the missing absorptive capacity [26]. Such innovation strategies by means of acquisition and consolidation are already occurring in the MN industry and may contribute to progressing to the final stage of convergence: maturation [7].

We argue that the result of food-pharma convergence into the MN industry is both supply (technology) and demand (consumer) driven. For example, technology has made it possible to reduce the volume of high-protein oral nutritional supplements (ONS) while simultaneously, due to a higher awareness of MN effectiveness, the demand for low-volume high-protein ONS is rising. Due to convergence of the supply (pharma) and demand (food) sides, a new MN value chain emerges. Value chain reconfiguration as a result of industry convergence may lead to the elimination of entire value chain steps or activities while other, value-added value chain activities may be introduced [61], [63].

**Figure 9:**
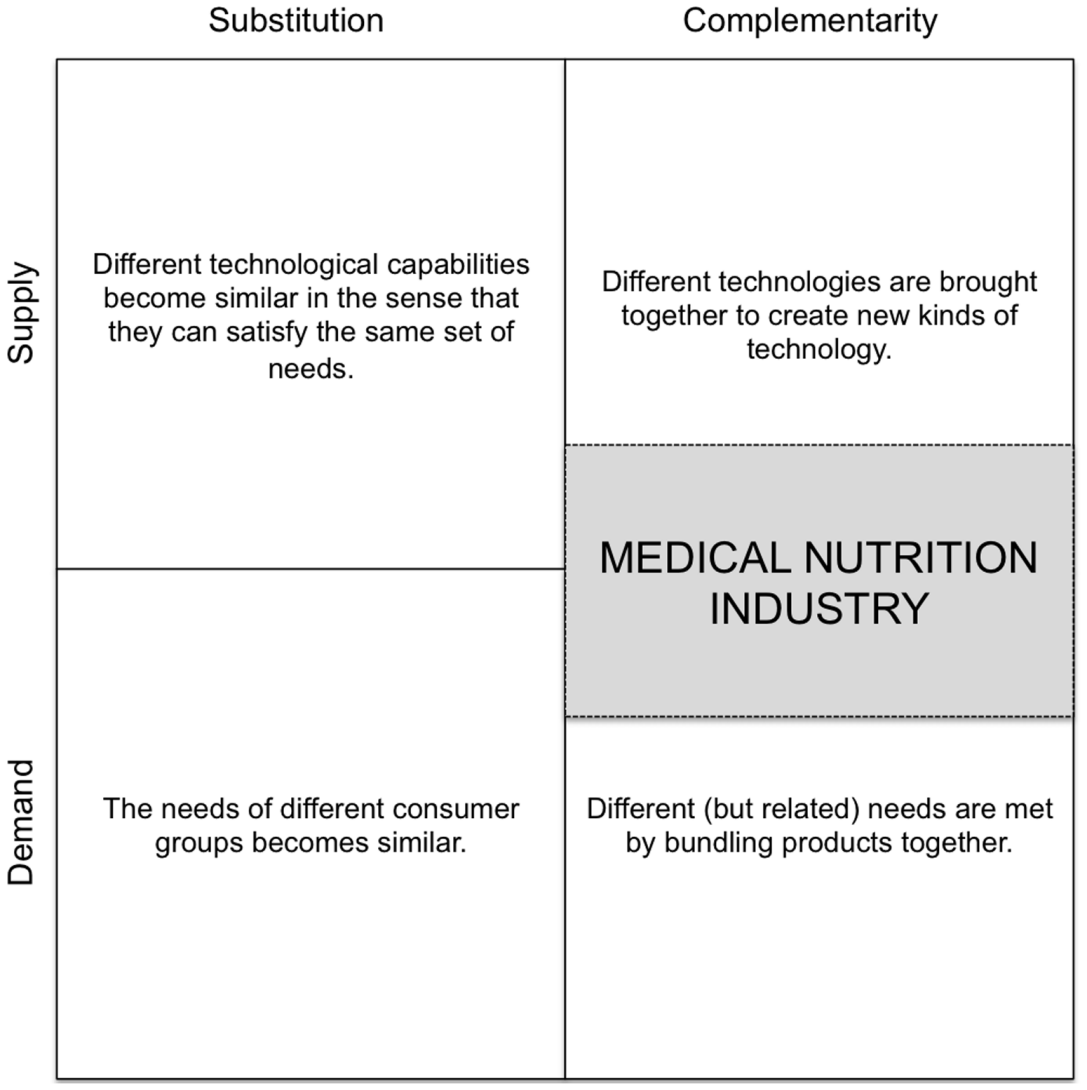
Categorization of MN industry convergence, adapted from [64].

In addition to diagnosing the MN industry to be in stage three of the industry convergence life cycle, the process of convergence in itself comes in two varieties; substitutive and complementary. Such a classification allows for characterization of the convergent industry. In the case of substitutive convergence, innovation leads to a phasing out of the two formerly discrete operating industries. Consequently; the added value of the complementary products combined is higher when compared to the individual components, thereby resulting in technological substitution from a consumer perspective (1+1 = 1). Complementary convergence is the process whereby previously unrelated products are bundled together to form a new combined and integrated class of product with added value for end-users (1+1 = 3) [61], [63]. In this case, the convergence between technologies results from technology fusion or by bundling exemplify complementarities [64]. The MN industry belongs to the second category in the view that MN replaces neither conventional foods nor pharmaceutical products (Figure 9).

Ultimately, additional research is required to understand the full impact of the MN industry within the context of the individual food and pharmaceutical industries. While this study focused on the use of patents to identify the stages of industry convergence, future research could focus on complementary data and methods for mapping the convergence process. One option may be to look at clinical research data by assessing to what extent these studies meet pharma industry standards. The MN industry offers a unique dataset for studying industry convergence and experimenting with tools on how this is best accomplished.

## References

[1] Eussen SR, Verhagen H, Klungel OH, Garssen J, van Loveren H, et al (2011) Functional foods and dietary supplements: products at the interface between pharma and nutrition. European Journal of Pharmacology 668 Suppl 1S2–9. 21816140 10.1016/j.ejphar.2011.07.008

[2] Phyllis Balch A (2010) Prescription for Nutritional Healing: Penguin Group (USA) Incorporated.

[3] Henry CJ (2010) Functional foods. Eur J Clin Nutr 64: 657–659. 20606685 10.1038/ejcn.2010.101

[4] Howlett J (2008) Functional Foods From Science to Health and Claims. ILSI Eur Concise Monogr: 1–36.

[5] van Kreijl CF, Knaap AGAC, van Raaij JMA (2006) Our food, our health: healthy diet and safe food in the Netherlands. 189–213.

[6] Bagchi D (2008) Neutraceutical and Functional Food Regulations. Elsevier: New York.

[7] Weenen TC, Pronker ES, Fernald KDS, Claassen E, Commandeur H (In press 2013) Bridging a pharma-like innovation gap in medical nutrition.

[8] El Sohaimy S (2012) Functional foods and nutraceuticals-modern approach to food science. World Applied Sciences Journal 20: 691–708.

[9] Weenen TC, Pronker ES, Commandeur HR, Claassen E (2013) Patenting in the European medical nutrition industry: Trends, opportunities and strategies. PharmaNutrition.

[10] Georgiou NA, Garssen J, Witkamp RF (2011) Pharma-nutrition interface: The gap is narrowing. European Journal of Pharmacology 651: 1–8. 21114994 10.1016/j.ejphar.2010.11.007

[11] Verhagen H, Vos E, Francl S, Heinonen M, van Loveren H (2010) Status of nutrition and health claims in Europe. Arch Biochem Biophys 501: 6–15. 20417175 10.1016/j.abb.2010.04.012

[12] Pronker ES, Weenen TC, Commandeur H, Claassen EH, Osterhaus AD (2013) Risk in vaccine research and development quantified. Plos One 8: e57755. 23526951 10.1371/journal.pone.0057755 PMC3603987

[13] Ernst H (1997) The use of patent data for technological forecasting: The diffusion of CNC-technology in the machine tool industry. Small Business Economics 9: 361–381.

[14] Gilbert L (1997) The consumer market for functional foods. Journal of Nutraceuticals, Functional and Medical Foods 1(3): 5– 21.

[15] Grunert KG, Bech-Larsen T, Bredahl L (2000) Three issues in consumer quality perception and acceptance of dairy products. International Dairy Journal 10: 575–584.

[16] Weststrate JA, van Poppel G, Verschuren PM (2002) Functional foods, trends and future. British Journal of Nutrition 88: S233–S235. 12495465 10.1079/BJN2002688

[17] Siro I, Kapolna E, Kapolna B, Lugasi A (2008) Functional food. Product development, marketing and consumer acceptance-A review. Appetite 51: 456–467. 18582508 10.1016/j.appet.2008.05.060

[18] Teratanavat R, Hooker NH (2006) Consumer valuations and preference heterogeneity for a novel functional food. Journal of Food Science 71: S533–S541.

[19] (MNI) MNI (2012) Oral Nutritional Supplements to Tackle Malnutrition Medical Nutrition International Industry. Available: http://www.medicalnutritionindustry.com/. Accessed 2013 August 23.

[20] von Delft S (2013) Inter-industry innovations in terms of electric mobility: Should firms take a look outside their industry? Letter from the Editor 10: 67.

[21] Hacklin F (2008) Management of Convergence in Innovation – Strategies and Capabilities for Value Creation Beyond Blurring Industry Boundaries. Physica-Verlag.

[22] Curran CS, Leker J (2011) Patent indicators for monitoring convergence - examples from NFF and ICT. Technological Forecasting and Social Change 78: 256–273.

[23] Duysters G, Hagedoorn J (1998) Technological convergence in the IT industry: the role of strategic technology alliances and technological competencies. International Journal of the Economics of Business 5: 355–368.

[24] Katz ML (1996) Remarks on the economic implications of convergence. Industrial and Corporate Change 5: 1079–1095.

[25] Prahalad CK (1998) Managing discontinuities: The emerging challenges. Research Technology Management 41: 14–22.

[26] Bröring S, Leker J (2007) Industry convergence and its implications for the front end of innovation: a problem of absorptive capacity. Creativity and Innovation Management 16: 165–175.

[27] Shi X, Adamic LA, Tseng BL, Clarkson GS (2009) The impact of boundary spanning scholarly publications and patents. PloS one 4: e6547. 19688087 10.1371/journal.pone.0006547 PMC2722725

[28] Bossink BAG (2004) Managing drivers of innovation in construction networks. Journal of Construction Engineering and Management-Asce 130: 337–345.

[29] Chang SB, Lai KK, Chang SM (2009) Exploring technology diffusion and classification of business methods: Using the patent citation network. Technological Forecasting and Social Change 76: 107–117.

[30] Karvonen M, Kässi T (2012) Patent citations as a tool for analysing the early stages of convergence. Technological Forecasting and Social Change.

[31] Williams J, Gannon F, O'Leary B, Ryan F (2009) Health LifeSciences in Ireland - An Enterprise Outlook. FORFÁS. Available: http://www.idaireland.com/news-media/publications/library-publications/external-publications/Health_ Life_Sciences_ in_Ireland.pdf. Accessed 2013 September 10.

[32] Lukach R, Plasmans J (2002) Measuring Knowledge Spillovers Using Patent Citations: Evidence from the Belgian Firm’s Data. CESifo Working Paper 754..

[33] Nemet GF, Johnson E (2012) Do important inventions benefit from knowledge originating in other technological domains? Research Policy 41: 190–200.

[34] Blackman M (1995) Provision of patent information: a national patent office perspective. World Patent Information 17: 115–123.

[35] Kim C, Lee H, Seol H, Lee C (2011) Identifying core technologies based on technological cross-impacts: An association rule mining (ARM) and analytic network process (ANP) approach. Expert Systems with Applications 38: 12559–12564.

[36] OECD (1994) Using patent data as science and technology indicators. Patent manual, OECD.

[37] Tseng FM, Hsieh CH, Peng YN, Chu YW (2011) Using patent data to analyze trends and the technological strategies of the amorphous silicon thin-film solar cell industry. Technological Forecasting and Social Change 78: 332–345.

[38] Choi C, Kim S, Park Y (2007) A patent-based cross impact analysis for quantitative estimation of technological impact: The case of information and communication technology. Technological Forecasting and Social Change 74: 1296–1314.

[39] Geum Y, Kim C, Lee S, Kim MS (2012) Technological Convergence of IT and BT: Evidence from Patent Analysis. Etri Journal 34: 439–449.

[40] Hu AGZ, Jaffe AB (2003) Patent citations and international knowledge flow: the cases of Korea and Taiwan. International Journal of Industrial Organization 21: 849–880.

[41] Trajtenberg M (1990) A Penny for Your Quotes - Patent Citations and the Value of Innovations. Rand Journal of Economics 21: 172–187.

[42] Yoon B, Park Y (2004) A text-mining based patent network: Analytical tool for high-technology trend. The Journal of High Technology Management Research 15: 37–50.

[43] Jaffe AB, Trajtenberg M (1999) International Knowledge Flows: Evidence From Patent Citations. Economics of Innovation and New Technology 8: 105–136.

[44] Csardi G, Strandburg KJ, Zalanyi L, Tobochnik J, Erdi P (2007) Modeling innovation by a kinetic description of the patent citation system. Physica a-Statistical Mechanics and Its Applications 374: 783–793.

[45] Dahlin KB, Behrens DM (2005) When is an invention really radical? Defining and measuring technological radicalness. Research Policy 34: 717–737.

[46] Borgatti SP, Everett MG, Freeman LC (2002) Ucinet for Windows: Software for Social Network Analysis. Harvard, MA: Analytic Technologies.

[47] Breschi S, Lissoni F, Malerba F (2003) Knowledge-relatedness in firm technological diversification. Research Policy 32: 69–87.

[48] EPO (2013) Cooperative Patent Classification (CPC). European Patent Office Available: http://www.epo.org/searching/essentials/classification/cpc.html. Accessed 2013 June 12.

[49] Johnstone N, Hascic I, Popp D (2010) Renewable Energy Policies and Technological Innovation: Evidence Based on Patent Counts. Environmental & Resource Economics 45: 133–155.

[50] Suzuki J, Kodama F (2004) Technological diversity of persistent innovators in Japan - Two case studies of large Japanese firms. Research Policy 33: 531–549.

[51] CPC Cooperative Patent Classification Cooperative Patent Classification European Patent Office, United States Patent andTrademark Office. Available: http://www.cooperativepatentclassification.org/index.html. Accessed 2013 June 12.

[52] Lee H, Kim C, Cho H, Park Y (2009) An ANP-based technology network for identification of core technologies: A case of telecommunication technologies. Expert Systems with Applications 36: 894–908.

[53] Jeong GH, Kim SH (1997) A qualitative cross-impact approach to find the key technology. Technological Forecasting and Social Change 55: 203–214.

[54] Enzer S (1972) Cross-Impact Techniques in Technology Assessment. Futures 4: 30–&.

[55] Schuler A, Thompson WA, Vertinsky I, Ziv Y (1991) Cross Impact Analysis of Technological Innovation and Development in the Softwood Lumber Industry in Canada - a Structural Modeling Approach. Ieee Transactions on Engineering Management 38: 224–236.

[56] Thorleuchter D, Van den Poel D, Prinzie A (2010) A compared R&D-based and patent-based cross impact analysis for identifying relationships between technologies. Technological Forecasting and Social Change 77: 1037–1050.

[57] Dernis H, Guellec D (2002) Using patent counts for cross-country comparisons of technology output. Special Issue on New Science and Technology Indicators 27: 129–146.

[58] Hall B, Jaffe A, Trajtenberg M (2001) The NBER patent citations data file: lessons, insights and methodological tools. National Bureau of Economic Research, Working Paper No 8498.

[59] Fürst P, Kuhn KS (2000) Amino-acid substrates in new bottles: implications for clinical nutrition in the 21st century. Nutrition 16: 603–606. 10906569 10.1016/s0899-9007(00)00321-x

[60] Broring S, Cloutier LM, Leker J (2006) The front end of innovation in an era of industry convergence: evidence from nutraceuticals and functional foods. R & D Management 36: 487–498.

[61] Weaver B (2007) Research proposal: Industry convergence-Driving forces, factors and consequences.

[62] Weenen TC, Pronker ES, Commandeur HR, Claassen E (2013) Barriers to innovation in the medical nutrition industry: A quantitative key opinion leader analysis. PharmaNutrition.

[63] Greenstein S, Khanna T (1997) What does industry convergence mean. Competing in the age of digital convergence: 201–226.

[64] Pennings JM, Puranam P (2000) Market convergence & firm strategies: towards a systematic analysis. Retrieved August 27: 2010.

[65] Curran CS, Broring S, Leker J (2010) Anticipating converging industries using publicly available data. Technological Forecasting and Social Change 77: 385–395.

